# Supplementary figures and images for: Isoflurane mediated neuropathological and cognitive impairments in the triple transgenic Alzheimer’s mouse model are associated with hippocampal synaptic deficits in an age-dependent manner
Source: PLoS One. 2019 Oct 10;14(10):e0223509. doi: 10.1371/journal.pone.0223509 (PMC6786564; doi:10.1371/journal.pone.0223509)

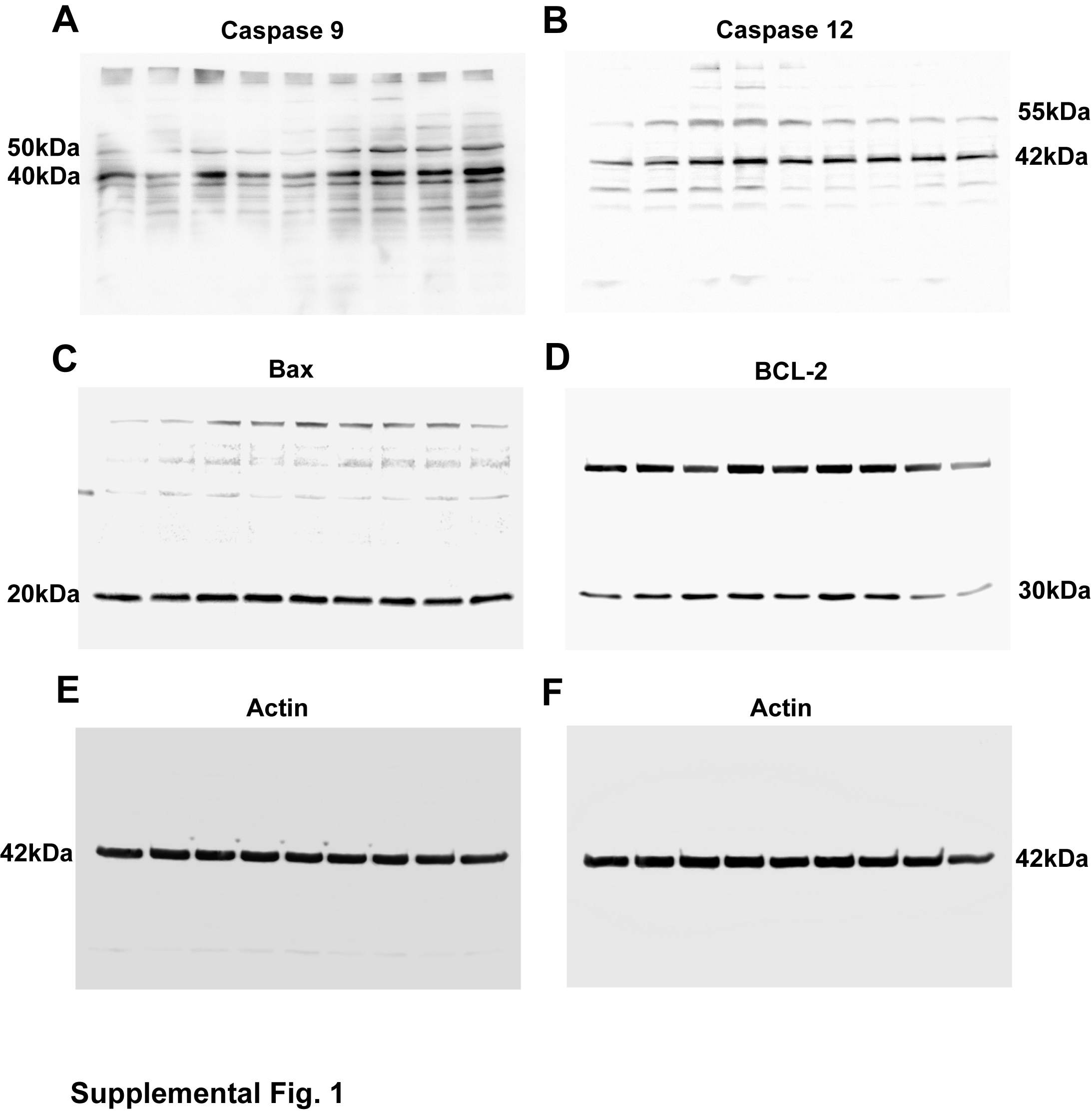

Supplement: S1 Fig — Representative blots of cleaved caspase 9 (A, 40kDa), caspase 12 (B, 42kDa), Bax (C, 20kDa), BCl-2 (D, 28kDa), and b-actin (E & F, 42kDa. Upper bands indicated in blots A & B by 50 (Caspase 9) and 55 kDa (caspase 12) are procaspase 9 and uncleaved caspase 12, respectively. (TIF) [file pone.0223509.s001.tif]

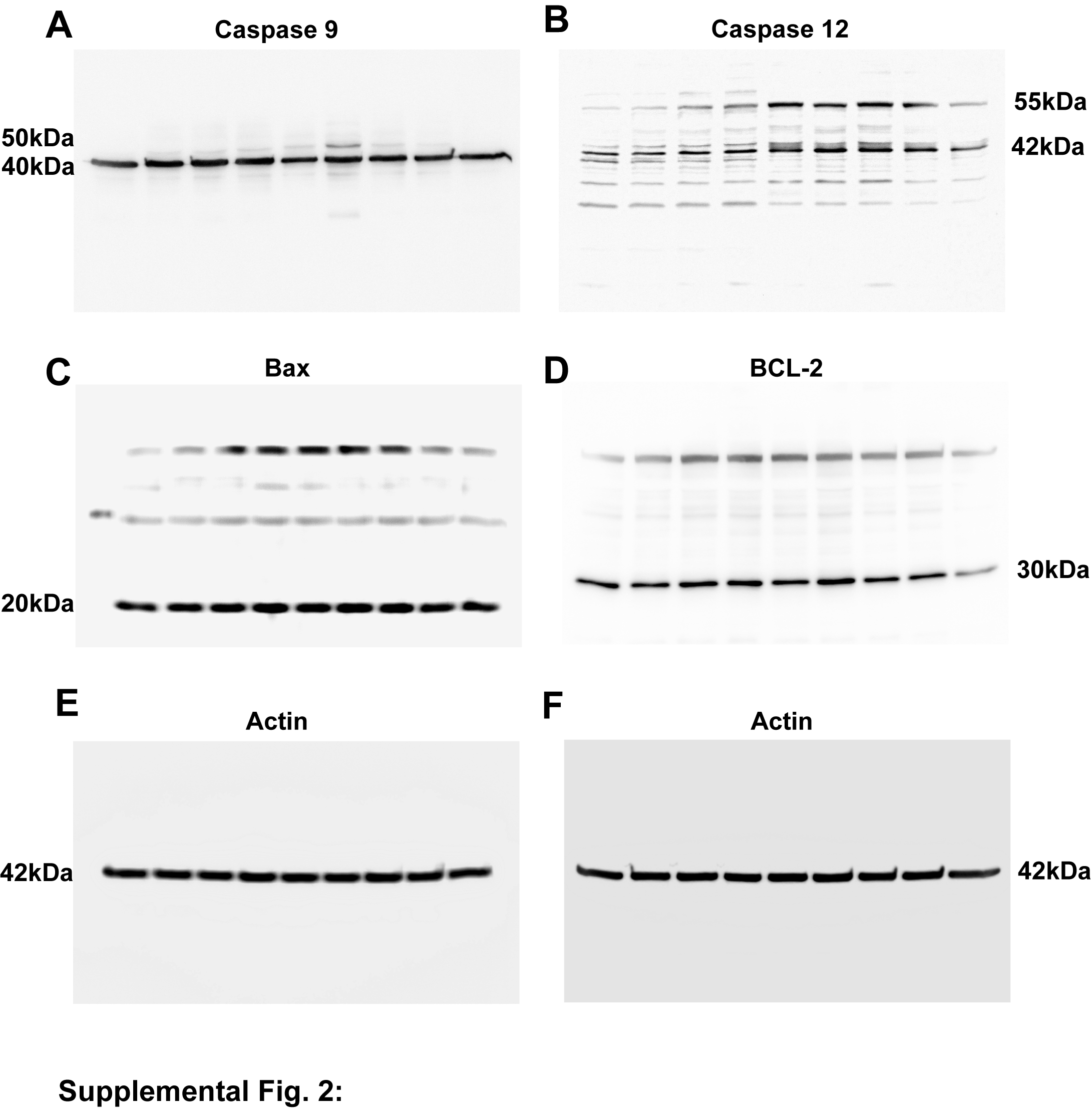

Supplement: S2 Fig — Representative blots of cleaved caspase 9 (A, 40kDa), caspase 12 (B, 42kDa), Bax (C, 20kDa), BCl-2 (D, 28kDa), and b-actin (E & F, 42kDa. Upper bands indicated in blots A & B by 50 (Caspase 9) and 55 kDa (caspase 12) are procaspase 9 and uncleaved caspase 12, respectively. (TIF) [file pone.0223509.s002.tif]
